# The effect of social interaction on decision making in emergency ambulance teams: a statistical discourse analysis

**DOI:** 10.1186/s12909-023-04091-w

**Published:** 2023-02-20

**Authors:** Murat Tekïn, İbrahim Uysal, Çetin Toraman, Canan Akman, Ayşen Melek Aytuğ Koşan, Emine Sevïnç Postaci

**Affiliations:** 1 Department of Medical Education, Çanakkale Onsekiz Mart University, Çanakkale, Turkey; 2 Department of First and Emergency Aid, Çanakkale Onsekiz Mart University, Çanakkale, Turkey; 3 Department of Emergency Medicine, Çanakkale Onsekiz Mart University, Çanakkale, Turkey

**Keywords:** Emergency Medical Services, Cognitive flexibility, Social Interaction, Statistical discourse analysis, Teamwork

## Abstract

**Introduction:**

This study examined the effects of social interaction, cognitive flexibility, and seniority on the correct response among emergency ambulance teams during case intervention.

**Methods:**

The research, structured with the sequential exploratory mixed method, was conducted with 18 emergency ambulance personnel. The approach process of the teams working on the scenario was video recorded. The records were transcribed by the researchers, including gestures and facial expressions. Discourses were coded and modeled with regression.

**Results:**

The number of discourses was higher in groups with high correct intervention scores. As the level of cognitive flexibility or seniority increased, the correct intervention score tended to decrease too. Informing has been identified as the only variable that positively affects the correct response to the emergency case, especially in the first period, which is directed toward case intervention preparation.

**Conclusion:**

Within the findings of the research, it is recommended that activities and scenario-based training practices that will increase the intra-team communication of the emergency ambulance personnel should be included in the medical education and in-service training.

**Supplementary Information:**

The online version contains supplementary material available at 10.1186/s12909-023-04091-w.

## Introduction

Pre-Hospital Emergency Medical Services (PH-EMS) cover the initial evaluation, emergency response, and appropriate transfer to the hospital, carried out by professional health personnel, who are transported to the scene by emergency ambulances and trained for such emergency assistance cases. This health service, which is taken to where the patient or injured is, directly affects the morbidity and mortality rates. Decisions made as a team in complex cases under stress and environmental factors by emergency ambulance personnel are vital for the served individuals [1].

Health personnel working in emergency health service stations work as a group in 24-hour shifts. In studies on patient safety, it has been accepted that team performance is essential to provide safe patient care [2]. Therefore, teamwork is the focal point of systems-based interventions to improve patient safety and medical education standards [3]. A group/team can be defined as two or more people who work together to achieve a specific goal, have task-specific competencies and job roles, use shared resources together, and communicate to adapt to rapid changes in coordination [4]. The common point of the groups is expressed as “the interaction of group members” [5].

Medical conditions such as injuries and traffic accidents, in which emergency assistance arises, are often complex situations requiring clinical reasoning. Intervening a problem as a team may be more effective than an individual effort. Many skills are used during this intervention. Of utmost importance among these skills is communication skills. Effective communication within the team will affect the clinical reasoning skills of the emergency ambulance team. The quality of the communication between the emergency ambulance team and the way they use their clinical reasoning skills while intervening crucial. The idea that ‘the group is more than the members that make up that group’ is one of the basic principles of social psychology [6]. Observational studies on teamwork behaviors related to effective clinical performance have identified communication, coordination, and leadership patterns that support effective teamwork [3].

Groups have various functions. The intimacy formed in the group meets the adaptation needs of the group members, tasks the group handles meet the achievement needs of the group members, and sociability in the group allows for meeting the identity-based needs of group members [7]. People feel and behave differently when doing things together, as if their individual minds become components of a “group mind.“ Much empirical evidence supports this metaphor on collaborative social interactions such as group problem-solving [6]. For example, the performance of a small group in solving problems may differ from the collective individual performance of all group members or the most productive group member working alone [8–11].

The communication component, one of the elements that support effective teamwork, can play a role that supports emergency teams’ clinical reasoning skills for working as a team. Clinical reasoning, on the other hand, is considered the cognitive process underlying clinical practice and is often referred to as problem-solving. While novice learners tend to have little and scattered knowledge, knowledge is organized and placed in more advanced structures and schemes with increasing experience. Studies reveal that clinical reasoning is a critical component of clinical competence [12, 13]. Clinical reasoning skills will make it easier for ambulance personnel to make decisions in the face of complex clinical situations and make the correct diagnosis and treatment direction possible. When people collaborate, they implement cognition loading and outsourcing [14]. Therefore, human behavior and mental processes differ not only quantitatively but also qualitatively between a group rather and an individual [6]. When ambulance teams intervene in emergency cases, how they use clinical reasoning skills on the case with the power of intercommunication is a determinant for correct intervention. There is no research on the effect of communication on clinical reasoning, which is one of the factors supporting effective teamwork in cases that require high clinical performance.

Regression analysis examines how variables before the event and discourse affect the final state in many situations where sequential discourses are experienced, such as during emergency response. Different studies that encode and digitize the discourses of group members and model them in regression analysis have taken their places in the literature. For example, Chiu and Khoo used regression analysis on solving problems in small groups in mathematics classes through the interaction between students’ certain characteristics and discourses in the process and showed less creativity in smaller groups in the mathematics lesson [15]. While Chiu showed how discourses affect the final situation in marital conflicts and examined the influence of discourse on preschool games [16]. Toraman and Karadağ used regression analysis to examine the effects of various variables (cognitive flexibility, school success, physics course success, sex, popularity, family income level) on high school students and the discourses formed in the communication between students during the problem-solving process [17].

This research examines the effects of communication, social interactions, clinical reasoning skills, and seniority characteristics of ambulance personnel on the correct case intervention.

## Method

The research was carried out with 18 ambulance personnel who volunteered to participate and allowed audio and video recordings with their informed consent. First, the speeches, gestures, and facial expressions of the personnel working as a team were recorded with video and audio recordings. These discourses were then coded. Finally, these discourses showing the quality of communication and cognitive flexibility, and seniority characteristics were modeled using regression modeling. Therefore, the research was structured with the sequential exploratory mixed method [18, 19].

## Participants

Participants were emergency ambulance personnel, who served as a team at the stations they served, worked with their own teams (in groups of three) on the cases given by the researchers. The researchers explained the purpose of the study and how it would be conducted to volunteer paramedics. Information on the group work being video recorded was provided. A total of 18 paramedics participated in the study, who signed the informed consent forms and approved the audio and video recording.

## Data collection tool

Within the scope of the research, data were collected on a case that allows paramedics working in emergency ambulances to work in groups and demonstrate their decision-making and communication skills. This process was video recorded, the team’s conversations and body languages while solving the case were written down, the conversations were coded, and the effect on the decision-making skills was analyzed.

The case was created by researchers and structured by expert control. Researchers included specialists in emergency medicine, medical education, and educational sciences. Afterward, the prepared case was presented to the opinion of four emergency medicine and three medical education specialists apart from the researchers. In line with the opinions received, corrections were made to the case, and its final form was given (Appendix 1).

The cognitive flexibility level of the emergency ambulance personnel participating in the research was determined by the Cognitive Flexibility Scale developed by Martin and Rubin [20] and adapted to Turkish by Altunkol for university students [21]. The scale is a Likert-type, 6-point scale consisting of 12 items. The relevant author presented the validity and reliability proofs for the Turkish adaptation of the scale. The scale consists of 12 items, has a single-factor structure, a minimum possible score of 12, and a maximum score of 72.

## Process

The research was completed following the procedures listed below:

- The approval of the ethics committee and relevant institutions was obtained to carry out the research.
- Before the group work, necessary explanations were made to paramedics, and expectations were explained. To increase validity and reliability, study participatory opportunities for paramedics when they wish to leave were clearly specified (honesty in informants), and only voluntary participants were included [22]. Of these, 18 paramedics who worked in emergency ambulances, volunteered to participate in the study, and gave consent for audio and video recording were included.
- Paramedics were divided into six groups of three team members working together. The simulated emergency intervention case (without the application on the real case) was given to the groups. The same case was given to the groups simultaneously, and interactions were recorded at six separate stations.
- In the simulation consisting of three stages, 10 min were given for each stage, adding up to a total of 30 min.
  1. In the first stage, as per the scenario, the information described by the patient’s relatives at the scene was presented to the teams by the command control center (see Appendix 1). At this stage, the teams were asked to discuss and write down their possible preliminary diagnoses. In addition, they were asked to write down the reasons for their preliminary diagnoses and the preparations they would make while going to the scene.
  2. In the second period, scene arrival, the incident was described, and the findings of the primary evaluation were given. The teams were then asked to discuss whether the probability of any preliminary diagnoses decreased after the information was presented and to write down the reasons.
  3. In the third period, secondary evaluation findings (thorough examination, sample, and vital parameters) of the case and examination results related to the case were given (see Appendix 1). With the last information given, they were asked to write down “the last diagnosis for the patient and why they thought about this diagnosis” and “interventions to be made for their diagnosis” by discussing in the team.
- Care was taken to obtain valid data per the purpose of the research. The whole process was recorded with video cameras. In the video camera recording, a professional camera and a microphone were used to clearly capture the voices of the paramedics in the group.
- For credibility, the researchers transcribed participants’ speeches in the video and audio recordings (including their gestures, mimics, and jokes) [23].
- Each team assigned one team member to read the case form. Discourses in which the case was read were coded as question reading and were excluded from the analysis.
- There were several discourses that could not be understood while listening to the audio recordings and transcriptions. These discourses were coded as ‘incomprehensible’ and excluded from the analysis. After the discourses of reading questions and excluding the incomprehensible conversations, the remaining 1640 discourses were coded and analyzed. Discourses were coded by the researchers. Coding was carried out as follows.

Paramedic 1: Preliminary diagnosis may be “arrest” due to sports injury, friends, because it’s a diving accident! (Informing)

Paramedic 2: Couldn’t it be MI too? (Asking Questions)

Paramedic 1: Command center told us he had a rash on his head. In other words, chest pain may be “arrest” due to MI or “arrest” due to trauma. Shall I write this on our answer sheet? (Guiding/Directing)

Paramedic 3: Okay, write. (Approving)

Paramedic 1: I’m writing sports injury. Arrest due to sports injury. (Suggesting)

Paramedic 2: Did he jump headfirst? (Asking Questions)

Paramedic 1: He dived, yes. (Responding)

Paramedic 3: First has stomach ache and palpitations. As a result, he jumps to relax. (Giving More Information)

Paramedic 2: Yes. (Approving)

Paramedic 3: Let’s not miss this, he does not go swimming directly into the sea. Meanwhile, he jumps into the sea to relax. His friends said, “He coughed by holding his chest while getting out of the water.“ Here he comes out of the water with his own means. (Informing)

Paramedic 1: Yes. (Approving)

Paramedic 3: It doesn’t seem like a very serious neck trauma. As long as he can get out of the water by himself. (Guiding/Directing)

Paramedic 1: There is mention of a rash on his forehead. (Guiding/Directing)

Paramedic 3: He also has a rash on his forehead. It may be related to diving; as you said, there may be an injury, but it is not very serious. (Informing)

- The discourse in which the text of the conversation was written was read. The contextual nature of each discourse in the video recording was examined. The semantic nature of each discourse has been examined. Codes for suggestions, approvals, rejections, guidances/directives, etc. were assigned.
- Discourses were coded in the research. Then, two of the 18 paramedics participating in the study were reached again. Their opinions were taken on whether the coding reflected what they said. This way, the participant verification strategy was used to provide credibility [23, 24].
- Discourses were coded independently by 5 experts, two of whom were emergency medicine specialists, one experienced in discourse analysis, two qualitative research specialists, and an educational scientist. These 5 coding experts were not included in the research and were experts in external evaluators. In this way, an external audit [19, 24, 25] was provided.
- Is the coding made consistent? Inter-rater reliability can be examined with Kripphendorff Alpha in the process performed by more than two raters assigning codes to more than two categories [26]. The qualitative codes (suggestion, approval, rejection, etc.) given to the discourses were converted into numerical codes, and the consistency between the codes was evaluated with the Krippendorff Alpha coefficient. The consistency level was determined as 0.93. According to the value obtained, it was decided that the coding consistency was high. These procedures were examined regarding inter-rater consistency, that is, reliability.
- A small amount of mismatched coding was detected between the external coders and the encodings performed by the researchers. A panel was held on which code would be given to these codes. In this panel, the codes were discussed, and the final code to be given was decided. This process utilizes the coding made by each researcher for other researchers’ usage to obtain reliable data (dependability) [27].
- Case-related responses of the six teams were scored according to clinical reasoning scoring criteria (the codes for the discourses and the clinical decision-making scores of the teams on the case) (see Appendix 1).
- As explained previously under the heading of data collection tools, the total scores of each participant from the cognitive flexibility scale were determined.
- All data were transferred to the statistical package program.

## Analysis of data

In this study, the variables in the data file and the data belonging to these variables can be summarized as follows:

- Discourses within the team while working on the case.

The case the teams are working on and the correct intervention process consists of three stages. In each stage, new information is added to the case, and the discussion gains a new dimension. For this reason, the discourses were divided into three periods: Discourses that took place in the first, second, and third periods.

The effect of discourses on the outcome (correct intervention) was included in the regression modeling for three periods separately. Multiple linear regression was used. The discourses included in the predictor variables were categorical (informing, responding, correcting, giving more information, ignoring, repeating, rejecting, approving, suggesting, etc.). Therefore, discourses were included in the regression model as dummy (dichotomous) variables. It is appropriate for categorical variables to be included in regression models as dummy variables [28, 29].

- Cognitive flexibility scores.

A cognitive flexibility scale was applied to each paramedic in each group. The total scores of each paramedic from the cognitive flexibility scale, obtained with the lowest 12 and the highest 72 points, were determined. Cognitive flexibility scores were continuous variables. In the regression modeling, cognitive flexibility scores were used as the group’s arithmetic mean of the three paramedics. While different regression models were created for the three periods, the cognitive flexibility group mean score was included in the model as a continuous variable.

- Seniority.

Seniority is the number of years worked by each paramedic in each group. Therefore, it is a continuous variable. The seniority of the three paramedics in each group was converted into an arithmetic mean. In the regression modeling, seniority was used as the arithmetic mean of the three paramedics in the group. While different regression models were created for the three periods, the seniority group mean was included in the model as a continuous variable.

- Success in case intervention.

Each group achieved a success score for the three intervention periods in the case (see Appendix 1). It is a continuous variable. These achievement scores were used as output variables in the regression models.

In the regression analysis, autocorrelation (multicollinearity) analysis was performed between predictors. In the regression models, the smallest Variance Inflation Factor (VIF) values for the variables were determined as 0.823, and the highest VIF values as 1.621. The obtained VIF values were very close to 1. If the VIF values calculated according to the literature are close to 1, it can be interpreted that there is no variance inflation factor and autocorrelation in the variables. VIF values above 5 are interpreted as variance inflation findings. Therefore, it was decided that there was no autocorrelation between predictors [30, 31].

## Results

The types and numbers of discourse occurring in each group for three periods were examined. In addition, the correct intervention scores (case example in Appendix 1 and the team’s score for correct intervention based on the information provided [outcome variable for this study]) obtained in the groups were also examined. The results are summarized in Table 1.

**Table 1:** Discourse types and clinical reasoning scores by periods

Period	Discourse	Group 1 f (%)	Group 2 f (%)	Group 3 f (%)	Group 4 f (%)	Group 5 f (%)	Group 6 f (%)
1	Informing	11 (13.4)	46 (24.2)	20 (22)	31 (21.4)	61 (36.3)	19 (21.3)
	Responding	2 (2.4)	----	2 (2.2)	2 (1.4)	----	----
	Correcting	----	4 (2.1)	----	3 (2.1)	4 (2.4)	3 (3.4)
	Giving More Information	4 (4.9)	----	----	----	----	----
	Ignoring	----	----	1 (1.1)	3 (2.1)	1 (0.6)	1 (1.1)
	Repeating	----	3 (1.6)	1 (1.1)	----	----	----
	Rejecting	2 (2.4)	7 (3.7)	4 (4.4)	8 (5.5)	5 (3)	6 (6.7)
	Approving	23 (28)	56 (29.5)	29 (31.9)	25 (17.2)	39 (23.2)	27 (30.3)
	Suggesting	15 (18.3)	57 (30)	22 (24.2)	54 (37.2)	37 (22)	22 (24.7)
	Asking Question	7 (8.5)	7 (3.7)	2 (2.2)	13 (9)	11 (6.5)	6 (6.7)
	Guiding/Directing	18 (22)	10 (5.3)	10 (11)	6 (4.1)	10 (6)	5 (5.6)
	**Total**	**82 (100)**	**190 (100)**	**91 (100)**	**145 (100)**	**168 (100)**	**89 (100)**
2	Informing	4 (7.7)	31 (35.2)	18 (23.1)	28 (28.9)	42 (42)	14 (31.8)
	Responding	1 (1.9)	----	----	1 (1)	----	----
	Correcting	----	1 (1.1)	2 (2.6)	5 (5.2)	1 (1)	4 (9.1)
	Giving More Information	5 (9.6)	----	----	----	----	----
	Ignoring	1 (1.9)	----	----	----	1 (1)	----
	Repeating	----	----	----	----	----	----
	Rejecting	1 (1.9)	3 (3.4)	1 (1.3)	2 (2.1)	1 (1)	3 (6.8)
	Approving	17 (32.7)	17 (19.3)	20 (25.6)	29 (29.9)	25 (25)	6 (13.6)
	Suggesting	6 (11.5)	18 (20.5)	22 (28.2)	19 (19.6)	17 (17)	9 (20.5)
	Asking Question	7 (13.5)	17 (19.3)	4 (5.1)	3 (3.1)	4 (4)	5 (11.4)
	Guiding/Directing	10 (19.2)	1 (1.1)	11 (14.1)	10 (10.3)	6 (6)	3 (6.8)
	**Total**	**52 (100)**	**88 (100)**	**78 (100)**	**97 (100)**	**100 (100)**	**44 (100)**
3	Informing	2 (4.9)	35 (25.4)	12 (27.3)	23 (27.1)	10 (17.9)	11 (21.2)
	Responding	1 (2.4)	----	----	1 (1.2)	----	----
	Correcting	----	----	----	1 (1.2)	----	3 (5.8)
	Giving More Information	3 (7.3)	----	----	----	----	----
	Ignoring	----	2 (1.4)	4 (9.1)	----	----	2 (3.8)
	Repeating	----	3 (2.2)	----	----	----	----
	Rejecting	----	6 (4.3)	2 (4.5)	1 (1.2)	2 (3.6)	4 (7.7)
	Approving	9 (22)	39 (28.3)	8 (18.2)	20 (23.5)	21 (37.5)	14 (26.9)
	Suggesting	----	29 (21)	13 (29.5)	27 (31.8)	13 (23.2)	14 (26.9)
	Asking Question	4 (9.8)	13 (9.4)	2 (4.5)	9 (10.6)	5 (8.9)	3 (5.8)
	Guiding/Directing	8 (19.5)	11 (8)	3 (6.8)	3 (3.5)	5 (8.9)	1 (1.9)
	**Total**	**41 (100)**	**138 (100)**	**44 (100)**	**85 (100)**	**56 (100)**	**52 (100)**
**General Total**		**175 (100)**	**416 (100)**	**213 (100)**	**327 (100)**	**324 (100)**	**185 (100)**
**Correct Intervention Scores**		**54**	**70**	**50**	**75**	**95**	**52**

**f**: Frequency, **%**: Percent

Correct intervention scores (case example in Aappendix 1 and the team’s score for correct intervention based on the information provided [outcome variable for this study]) also increased in groups where the number of discourses increased. It was determined that the group with the highest correct intervention score used informative discourse more than the other groups in the first and second periods. The discourse numbers of the groups with low correct intervention scores were below 200 and lower than those with high scores. A cognitive flexibility scale was applied to each paramedic in each group. The total scores of each paramedic from the cognitive flexibility scale, obtained with the lowest 12 and the highest 72 points, were determined. In the regression modeling, cognitive flexibility scores were used as the group’s arithmetic mean of the three paramedics. In addition, the cognitive flexibility levels and seniority of the paramedics in the groups were examined. The results are summarized in Table 2.

**Table 2:** Cognitive flexibility scores and seniority of paramedics in the groups

Groups	Team Member	Cognitive Flexibility	Seniority	Group Cog. Flexibility	Group Seniority	Correct Intervention Scores
				Mean (S. Deviation)	Mean (S. Deviation)	
1	Team member 1 (Female)	52	10	56.78 (4.17)	11.23 (2.12)	54
	Team member 2 (Male)	60	14			
	Team member 3 (Male)	61	9			
2	Team member 4 (Female)	61	10	64.98 (3.73)	9.11 (0.88)	70
	Team member 5 (Male)	67	8			
	Team member 6 (Male)	70	9			
3	Team member 7 (Female)	52	16	52.21 (1.38)	13.36 (2.53)	50
	Team member 8 (Female)	51	15			
	Team member 9 (Female)	54	10			
4	Team member 10 (Female)	43	10	49.41 (4.54)	12.19 (2.48)	75
	Team member 11 (Female)	55	10			
	Team member 12 (Male)	48	15			
5	Team member 13 (Male)	57	8	53.19 (7.21)	10.72 (3.04)	95
	Team member 14 (Male)	59	9			
	Team member 15 (Female)	43	15			
6	Team member 16 (Male)	54	14	57.73 (2.51)	10.59 (2.32)	52
	Team member 17 (Female)	58	8			
	Team member 18 (Male)	60	10			

The lowest possible score on the cognitive flexibility scale was 12, and the highest score was 72. The data showed that correct intervention scores did not increase proportionally with higher cognitive flexibility and seniority. Therefore, cognitive flexibility, seniority, and the level of predicting the correct intervention in the discourses formed in the group were examined separately in each period. Appendix 1 presents a case in which paramedic groups will be intervened simulatively. The case intervention consisted of three periods. Additional information is provided to the team at the beginning of each period. The first period is mostly related to the preliminary diagnoses of the cases and the reasons for these preliminary diagnoses and physical examinations. The second period is about reaching a more probable definitive diagnosis than preliminary diagnoses with new information given. The third period is about confirming the possible diagnosis reached with the new information given and the definitive diagnosis. The structure of the three periods is in the form of tasks within a task. The duties of paramedics also vary. For this reason, the regression modeling of the three periods was done separately. Because cognitive flexibility, seniority, and the nature of discourses (informing, responding, correcting, giving more information, ignoring, repeating, rejecting, approving, suggesting, etc.) may have different effects on reaching the right result, depending on the necessity of the task in the intervention. The results of the regression analysis performed are summarized in Table 3.

**Table 3:** The level of cognitive flexibility, seniority, and discourse for predicting the correct intervention in three different periods

Period	Predictor Variables	B (95% CI)	t	p	R	R^2^	Model ANOVA F (p)
1	Responding	-12.11 (-24.22/0.01)	-1.96	0.051	0.38	0.14	10.48 (< 0.0001)
	Correcting	2.88 (-5.21/10.97)	0.69	0.485			
	Giving More Information	-10.38 (-25.20/4.445)	-1.37	0.170			
	Ignoring	0.44 (-11.69/12.56)	0.07	0.944			
	Repeating	-2.66 (-17.44/12.12)	-0.35	0.724			
	Rejecting	-1.95 (-7.51/3.61)	-0.69	0.491			
	Approving	-1.38 (-4.29/1.53)	-0.93	0.351			
	Asking Questions	0.37 (-4.40/5.14)	0.15	0.879			
	Guiding/Directing	-5.32 (-9.64/-1.00)	-2.42	**0.016**			
	Seniority	-2.25 (-2.79/-1.71)	-8.23	**< 0.0001**			
	Flexibility	-0.89 (-1.08/-0.70)	-9.18	**< 0.0001**			
2	Responding	-15.93 (-37.21/5.35)	-1.47	0.142	0.41	0.17	8.29 (< 0.0001)
	Correcting	-10.60 (-19.27/-1.93)	-2.40	**0.017**			
	Giving More Information	-13.39 (-27.04/0.25)	-1.93	0.054			
	Ignoring	-2.82 (-24.05/18.41)	-0.26	0.794			
	Rejecting	-0.15 (-8.53/8.24)	-0.03	0.973			
	Approving	-3.38 (-7.18/0.42)	-1.75	0.081			
	Suggesting	-4.72 (-8.76/-0.68)	-2.29	**0.022**			
	Asking Questions	-7.41 (-12.78/-2.05)	-2.72	**0.007**			
	Guiding/Directing	-7.92 (-13.26/-2.58)	-2.91	**0.004**			
	Seniority	-2.65 (-3.33/-1.98)	-7.75	**< 0.0001**			
	Flexibility	-0.89 (-1.15/-0.63)	-6.66	**< 0.0001**			
3	Responding	-5.98 (-24.62/12.66)	-0.63	0.529	0.35	0.12	4.70 (< 0.0001)
	Correcting	-14.85 (-28.16/-1.54)	-2.19	**0.029**			
	Giving More Information	-16.18 (-31.46/-0.89)	-2.08	**0.038**			
	Ignoring	-13.08 (-22.67/-3.48)	-2.68	**0.008**			
	Repeating	3.33 (-12.09/18.74)	0.42	0.672			
	Rejecting	-3.31 (-10.51/3.88)	-0.91	0.366			
	Suggesting	-3.73 (-7.26/-0.21)	-2.08	**0.038**			
	Asking Questions	-0.55 (-5.56/4.46)	-0.21	0.830			
	Guiding/Directing	-3.44 (-8.75/1.87)	-1.27	0.204			
	Seniority	-1.94 (-2.63/-1.26)	-5.56	**< 0.0001**			
	Flexibility	-0.71 (-0.95/-0.47)	-5.72	**< 0.0001**			

In the regression analysis, “Informing” was determined as the reference group, and other discourses will be interpreted according to “Informing.“ The results showed that guiding/directing, cognitive flexibility, and seniority negatively predicted correct intervention in the first period. According to informing, the insignificance of discourses other than guiding/directing indicates that the reference group informing is a positive predictor of correct intervention. In the second period, the analysis showed that correcting, suggesting, asking questions, guiding/directing, cognitive flexibility, and seniority negatively predicted correct intervention. The analysis results for the third period showed that correcting, giving more information, ignoring, suggesting, cognitive flexibility, and seniority negatively predicted correct intervention in this period.

When the distribution of discourses according to the groups in Table 1, cognitive flexibility, seniority, and correct intervention scores obtained by the groups in Table 2, and the regression analyses in Table 3 are examined together, the following conclusion can be drawn. Different features may cause different effects at different stages of the intervention. For example, “correcting” worked as a variable that had a positive effect in determining possible preliminary diagnoses and a negative effect in the transition from possible preliminary diagnoses to a more probable diagnosis and in the final definitive diagnosis stages. On the other hand, cognitive flexibility was a variable with a negative effect in all three periods. Cognitive flexibility enables individuals to easily switch to lateral thoughts and see alternatives when faced with a new problem. Perhaps it is more effective to think with template information when faced with a case. However, this situation contradicts the fact that seniority has a negative effect. Because seniority seems to support approaching cases as more templates.

## Discussion

This study examined the effects of communication, interaction, clinical reasoning skills, and seniority characteristics of ambulance personnel who make critical decisions about human health in complex cases and respond as a team to emergency cases on the correct intervention. This is the first study in the literature to discuss how intra-team communication of ambulance personnel affects clinical reasoning skills. In addition, the high number of discourses in groups with high correct intervention scores, which emerged in the research findings, reveals the positive effect of the formation of an intense communication environment on the correct intervention. Determining the level of cognitive flexibility and seniority as variables that negatively affect the correct response to emergency cases were the findings that made this study unique.

One of the methods frequently used in the evaluation of group dynamics is the scenario-based observation method [32]. Different groups of healthcare providers perceive the quality of teamwork differently. Studies investigating the teamwork perceptions of clinicians show that variables such as leadership quality and openness of communication are generally evaluated positively [33–36]. In addition, observational studies have revealed weak to moderate relationships between ratings of teamwork skills and measures of technical and clinical performance [37–40]. Experimental research shows that system improvements [41] and specific team training interventions, such as formal practices to strengthen communication and relationships between healthcare providers, have the potential to increase clinicians’ awareness of these issues [42]. Although our research with ambulance personnel parallels the literature, it has been concluded that intra-team communication positively affects clinical reasoning and reaching the correct diagnosis in a case-specific manner.

In the study by Lingard et al. (2005), three video-based scenarios for the tension level, responsibility for creating, and resolving the tension of three professions working together as a surgeon, nurse, and anesthesiologist were evaluated independently. Although the tension levels of the three professions working together are similar, it has been revealed that each profession evaluates the responsibility of creating and solving tension differently and generally evaluates their profession as relatively less responsible than the others [43]. Since pre-hospital emergency health services take place where crisis situations, communication barriers, and communication accidents occur at a high rate, the personnel providing the service have a great responsibility to ensure effective communication in suppressing the crisis [44]. Ambulance teams show a different approach from other occupational groups by focusing on the emergency response as a team facing environmental stress factors, complex cases, and problems. In our study with the emergency ambulance team members, who focused on effective and quality service instead of evaluating the professions separately, it was revealed that the multiplicity of discourses in the group is important for the correct response to emergency cases. The high level of interaction of the group supported the high score obtained from the correct intervention. The number of discourses was higher in groups with high correct intervention scores. In addition, regression analysis showed that guiding/directing, cognitive flexibility, and seniority negatively predicted correct intervention in the first period.

Individuals with cognitive flexibility are expected to be aware of their options, cope effectively with new and difficult situations, produce alternative thoughts and ideas, and adapt to new situations. The increase in the seniority of the individuals in their profession takes them from apprenticeship to the level of expertise, teaches them to cope with an ever-expanding environment and increasing stimuli, and enables them to take the appropriate cognitive position in the face of the problem. The development of these skills, along with increasing seniority in the profession, is supported by cognitive flexibility [45]. It is thought that there will be an increase in the cognitive flexibility levels of the personnel involved in providing pre-hospital emergency health services with the experience they have gained in their professional lives. However, Uysal et al. revealed that professional experience (seniority) in emergency ambulance personnel does not make a significant difference in cognitive flexibility, deep or surface learning approach [1]. This study determined cognitive flexibility level and seniority as variables that negatively affect emergency response. As the level of cognitive flexibility or seniority increased, the correct intervention score tended to decrease. This result can be explained by the fact that the number of preliminary diagnoses is lower in groups with higher seniority and cognitive flexibility scores, and they resort to shortcuts to reach a definitive diagnosis.

Clinical reasoning is the information integration and synthesis from a clinical case with the knowledge and experience of healthcare professionals, and it’s used in the patient’s diagnosis and in the management of the patient [46]. Personnel with good communication skills in emergency ambulances, where many problems may arise, contribute to increasing the efficiency of the service [44]. The team leader working in emergency ambulances tends to present the emergency response without wasting time by focusing on the preliminary diagnoses so that they can make a quick decision according to the case’s urgency. In the study, discourse types showing the quality of interaction were either not significant on the correct response to the emergency case in the regression equation or were determined as significant variables in the negative effect. Only informing has been identified as a variable that positively affects the correct response to the emergency case, especially in the first period, which is directed toward case intervention preparation. This may be related to the transition process described above.

As in similar studies, some limitations in our study may affect the interpretation of our findings. Worldwide, emergency health services are provided based on various models and with teams of different members. In this respect, future research can contribute to increasing the generalizability of the results obtained in this research by working with teams providing emergency health services in different countries and health professionals from different seniorities.

Within the research findings, the decisions made as a team in complex cases under stress and environmental factors are vital for the individuals who receive service. Therefore, we recommend that activities and scenario-based training practices that will increase the intra-team communication of the ambulance personnel working as a team for 24 h should be included in the in-service training.

## Electronic supplementary material

Below is the link to the electronic supplementary material.

Supplementary Material 1

## Data Availability

The datasets used during the study are available from the corresponding author upon reasonable request. The datasets generated during and analyzed during the current study are not publicly available. The research data sets generated and analyzed during the current study are not publicly available due to the confidentiality announcement made on the participants but are available from the corresponding author upon reasonable and ethical request. We, as authors, hereby confirm that all methods were performed following the relevant guidelines and regulations stated in the Declaration of Helsinki.
