# Supplementary material for: The effect of social interaction on decision making in emergency ambulance teams: a statistical discourse analysis
Source: BMC Med Educ. 2023 Feb 20;23:121. doi: 10.1186/s12909-023-04091-w (PMC9942383; doi:10.1186/s12909-023-04091-w)
Supplement: Supplementary file 1 — Supplementary Material 1 [file 12909_2023_4091_MOESM1_ESM.docx]

**Appendix 1**

*Period 1 (Duration 10 minutes)*

Information given to the team;

On Sunday, at 15:00, a notice came to the emergency ambulance station you were on duty.

The command control center reported the case to you as “43-year-old male patient, the scene of the medical case is a public beach”. You were on your way to the case and the command control center has given you the following additional information by phone;

The patient's friends at the scene said that they came to the beach early in the morning to swim and sunbathed for a long time. They said that they had breakfast early in the morning and never ate anything else. His friends said that Hasan felt bad due to stomach pain and palpitations and that he decided to go into the water to regain himself. His friends rushed to his aid when they saw that Hasan ran from the pier and jumped into the water, then coughed by holding his chest and had a rash on his forehead as he got out of the water. When they came to him, Hasan told his friends that he felt bad, had pain in his chest and had difficulty in breathing. When he came to the shore with the help of his friends, exhausted, their friends who could not carry Hasan, who they held in their arms, laid him on the beach and called the emergency ambulance. As a team, you set out from the emergency station for this case.

**What were your possible preliminary diagnoses for this patient, what were the reasons that led you to this preliminary diagnosis, what equipment would you take with you when you first arrived at the scene, and what kind of intervention plan would you make?**

*Expected Responses*

Trauma, Acute Coronary Syndrome, Tachyarrhythmia, Respiratory distress/Acute pulmonary edema, Cerebrovascular accident, Diabetic emergency, Pulmonary Embolism, Peptic Ulcer, Heat stroke and Aneurysm pre-diagnoses; the rationale for these diagnoses, necessary team preparations and necessary equipment to prepare will be written in full. A total of 103 points can be obtained from the section.

*Period 2 (Duration 10 minutes)*

When you reached the scene, the patient was laid on the beach and a crowd gathered around him. When you see the patient, his body is wet, with a 1 cm abrasion in the frontal region and cold-pale skin. You secured the scene, and when you verbally warned the patient, he answered you half-consciously. When you asked the people around how it happened, they said that after getting out of the water, he felt nauseous, dizzy, and had a pressing pain in his back and chest. His best friend, Ali, says that Hasan smokes 1 pack of cigarettes a day, has high blood pressure, has diabetes, is not allergic to anything they know, they ate only a bagel in the morning and drank tea together, he has not had any surgery before, Ali said that Hasan took Fludex (Hypertension medicine) in the morning after breakfast. Meanwhile, Hasan, who called you tiredly, said that he felt pressure in his chest and had difficulty in breathing.

***In line with these new findings and information, have there been any changes in the preliminary diagnoses you thought for the patient? Which has become a priority? Why? Write your thoughts on the table with reasons.***

*Expected Responses*

Each correct spelling of whether the probability increases, decreases or remains the same is given a “1” point. “1” point is given for each correct reason. 38 points can be obtained from this section.

*Period 2 (Duration 10 minutes)*

**The following findings were detected in the patient whose 2nd evaluation was completed by you.**

T.A: 110/60 mmHg, Heart rate: 90/min., Respiration Rate: 24/min., SPO^2^: %92, Blood Sugar: 70 gr/dl

Body Temperature: 36 ⁰C, EKG: As given below, 1 cm Abroation in the frontal region, Pupillary: Isochoric, Has light reflex, No bleeding or fluid from nose, ear and mouth, Trachea midline, Jugular veins are normal, There are no abnormal features in the head-to-toe and system examinations., EKG finding; ST segment depression (not elevation) in the septal and anterior precordial leads.

***In line with these findings, what is your diagnosis/diagnoses for the patient, explain with reasons?***

***Explain your approach and intervention to the patient.***

*Expected Responses*

“Posterior MI” should be written as the final diagnosis (5 points). ECG finding for the reason, Chest Pain (1), - Stomach pain and back pain (1), Dyspnea accompanying pain (1) Chest pain (1), Palpitation (1).

- Manual cervical immobilization on suspicion of trauma (1)

-Cervical and vertebral stabilization by reaching the backboard and cervical color (1)

-Oxizenization at 6-8 lt/min concentration due to hypoxia (1)

-Double vascular access with at least 18G diameter cannula with the possibility of emergency drug administration (1)

-ECG/Monitorization (1)

-Acetylsalicylic acid 300 mg sublingual (if conscious state permits) (1)

- Effortless transfer to a suitable hospital with angiography (1)

- Since the systolic blood pressure is above 90 mmHG, I consider giving isordil. (1)

Maximum points that can be obtained: 18 points.
